# Potentially inappropriate polypharmacy is an important predictor of 30-day emergency hospitalisation in older adults: a machine learning feature validation study

**DOI:** 10.1093/ageing/afaf156

**Published:** 2025-06-06

**Authors:** Robert T Olender, Sandipan Roy, Prasad S Nishtala

**Affiliations:** Department of Pharmacy and Pharmacology, University of Bath, Claverton Down, Bath BA2 7AY, UK; Department of Mathematical Sciences, University of Bath, Bath, UK; Department of Pharmacy and Pharmacology & Centre for Therapeutic Innovation, University of Bath, Bath, UK

**Keywords:** artificial intelligence, machine learning, decision tree, predictive modelling, hospitalisation, older people

## Abstract

**Background:**

Machine learning (ML) models in healthcare are crucial for predicting clinical outcomes, and their effectiveness can be significantly enhanced through improvements in accuracy, generalisability, and interpretability. To achieve widespread adoption in clinical practice, risk factors identified by these models must be validated in diverse populations.

**Methods:**

In this cohort study, 86 870 community-dwelling older adults ≥65 years from the UK Biobank database were used to train and test three ML models to predict 30-day emergency hospitalisation. The three ML models, Random Forest (RF), XGBoost (XGB), and Logistic Regression (LR), utilised all extracted variables, consisting of demographic and geriatric syndromes, comorbidities, and the Drug Burden Index (DBI), a measure of potentially inappropriate polypharmacy, which quantifies exposure to medications with anticholinergic and sedative properties. 30-day emergency hospitalisation was defined as any hospitalisation related to any clinical event within 30 days of the index date. The model performance metrics included the area under the receiver operating characteristics curve (AUC-ROC) and the F1 score.

**Results:**

The AUC-ROC for the RF, XGB and LR models was 0.78, 0.86 and 0.61, respectively, signifying good discriminatory power. The DBI, mobility, fractures, falls, hazardous alcohol drinking and smoking were validated as important variables in predicting 30-day emergency hospitalisation.

**Conclusions:**

This study validated important risk factors for predicting 30-day emergency hospitalisation. The validation of important risk factors will inform the development of future ML studies in geriatrics. Future research should prioritise the development of targeted interventions to address the risk factors validated in this study, ultimately improving patient outcomes and alleviating healthcare burdens.

## Key Points

- Emergency hospitalisation carries additional risks for older adults ≥65 years.
- Risk factors identified by machine learning models must be validated in diverse populations.
- Drug Burden Index (DBI) is an important risk factor for 30-day emergency hospitalisation.

## Introduction

Emergency hospitalisation carries a high risk of complications [1] for older adults, such as functional decline [2, 3], cognitive impairment [4], hospital-acquired infections [5], delirium [6, 7] and readmission [8]. Machine learning (ML) approaches have been shown to predict clinical outcomes accurately [9], including but not limited to hospital admission [10], mortality [11], and heart failure-related hospitalisations [12]. However, ML suffers from concerns regarding interpretability and generalisability [13]. Risk factors identified using these models must be validated in other datasets and clinical settings.

In our previously published study, three ML models were developed and optimised for predicting 30-day hospitalisation in the International Resident Assessment Instrument—Home Care (InterRAI-HC), a dataset concerning community-dwelling older adults aged ≥65 years with complex care needs from New Zealand [14]. The study found that the Drug Burden Index (DBI) and alcohol consumption are potentially modifiable risk factors associated with 30-day hospitalisation. The DBI, validated in multiple care settings, serves as a measure of potentially inappropriate polypharmacy [15, 16]. DBI assesses the cumulative risk of medication-related exposure that could lead to functional impairment in older adults [17]. Recognition of DBI as an important factor for predicting hospitalisation could reduce the downstream patient risk associated with that prescribing by implementing a deprescribing response. High DBI has been linked to poor clinical outcomes [18], and while DBI reduction may lower emergency hospitalisation risk, deprescribing DBI drugs remains challenging and has seen limited success in randomised controlled trials [19] and recent meta-analysis [20]. However, deprescribing was also shown to be effective in a study by Nishtala *et al.*, where the group reported that DBI drug dose reduction resulted in significantly decreased DBI [21]. Fujita *et al.*, found that following a comprehensive intervention, the proportion of patients with at least one DBI-contributing medication ‘stopped/reduced’ increased from 29.9% to 37.5% [22]. In a study by Goldberg *et al.*, pharmacist medication review in four Emergency Departments (ED) in Spain showed no statistical significance in reducing admissions; however, the results indicated that the recommendations made were essential to reducing healthcare utilisation at two EDs at the 3-month mark [23]. Lowering the DBI appears to be a promising initiative for lowering 30-day hospitalisation risk; however, currently, there are no studies to definitively link DBI deprescribing to improved clinical outcomes [18, 20].

The UK Biobank, a biomedical database containing data on 500 000+ participants aged 40–69 from the UK, represents a different population with varying levels of comorbidity, data collection methodologies, and biases compared to the InterRAI. Validation of previously identified risk factors for 30-day emergency hospitalisation in the UK Biobank gives clinicians confidence that results are generalisable. Additionally, external validation may identify additional risk factors for 30-day-emergency hospitalisations within the UK Biobank dataset. The hypothesis was that important emergency hospitalisation risk factors identified in InterRAI would also be important in the UK Biobank. Thus, this study aimed to validate previously identified important risk factors associated with 30-day emergency hospitalisations using three ML models deployed on the UK Biobank data.

## Methods

The Academic Ethics and Integrity Committee at the University of Bath has approved this project (Form No: 6738). The current study followed the TRIPOD-AI checklist (Transparent Reporting of a multivariable prediction model for Individual Prognosis Or Diagnosis) [24], which can be seen in Supplementary Appendix 1.

### Data source: UK Biobank

The UK Biobank is a prospective cohort of 500 000+ community-dwelling volunteer participants, mostly aged between 40 and 69, assessed between 2006 and 2010 in England, Wales, and Scotland. Participant data includes demographic and lifestyle information, baseline physical examination results, and primary and secondary data from several healthcare system providers. New data are uploaded into the database regularly. More details concerning the UK Biobank and how to access the datasets can be found at www.ukbiobank.ac.uk/.

### Differences between UK Biobank and InterRAI

In the current study, results from an ML analysis of the UK Biobank dataset are compared and contrasted to a previously published study that utilised the InterRAI dataset [14]. Some notable differences between the UK Biobank and InterRAI are summarised in Supplementary Appendix 2. Most notably, the InterRAI concerns participants with complex care needs, whereas the UK Biobank concerns a healthier cohort of volunteer participants. This is further emphasised by the hospitalisation rate of 0.82% and 20.12% for the UK Biobank and InterRAI, respectively. Additionally, the UK Biobank is concerned with a significantly younger cohort, with a mean age at recruitment of 66.8, compared to 82.5 in the InterRAI. Finally, the two datasets have different data collection methodologies, InterRAI being retrospective and UK Biobank prospective.

### Study population and study design

**Figure 1:**
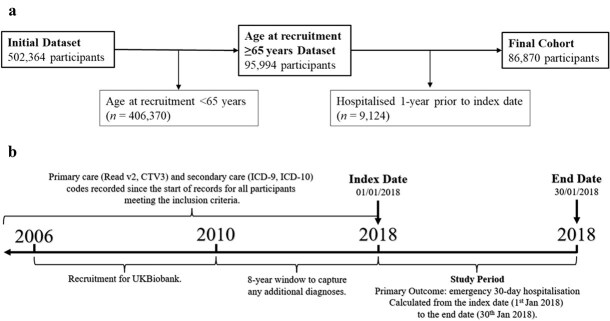
(a) Participant selection process. (b) Study design flowchart. *Note*. (a) Patients hospitalised 1 year before the index date were excluded from the analysis to account for healthy user bias. (b) CTV, clinical terms version; ICD, International Classification of Diseases.

In this cohort study, 95 994 participants with an age at recruitment 65–73 years were identified from the entire UK Biobank dataset. The index date was set as 1st January 2018, allowing additional diagnoses to be captured between 2010 and 2018. A total of 9124 participants hospitalised 1 year before the index date were excluded to account for healthy user bias, resulting in a final cohort of 86 870 participants. No further exclusions were made. A total of 715 participants had an emergency hospital admission within 30 days of the index date. A full demographic breakdown can be seen in Supplementary Appendix 3, with a correlation matrix in Supplementary Appendix 4. It should be noted that while the UK Biobank study considered participants aged 40–69 years, 2415 participants did not undergo assessment until they were aged 70, and a further 7 participants aged 71–73 years. These patients were included in the analysis. The participant selection process can be seen in Figure 1a. The study design is visualised in Figure 1b.

### Defining 30-day emergency hospitalisation

The study’s primary outcome was 30-day emergency hospitalisation, defined as any emergency admission within 30 days of the index date, 1st January 2018. Type of admission included but was not limited to Accident and Emergency (A&E), general practitioner referrals, and A&E transfers. Elective admissions were not included. Emergency admission data was obtained from the ‘hesin’ entity of the UK Biobank Research Analysis Platform (UKB-RAP).

### Defining independent variables

The predictors of 30-day emergency hospitalisation in the current study were chosen to match the InterRAI study dataset and were supplemented with several additional comorbidities, known risk factors for hospitalisation, geriatric factors, and demographic information. The variables included in the current study are available in most healthcare databases, and most can be obtained directly from the patient. Demographic information was categorised into factors. The Townsend deprivation, initially a continuous variable, had 68 missing values, imputed using mean imputation and then split into four quantiles. Twenty-five medical conditions were defined as binary factors, ‘1’ signifying history of a medical condition and ‘0’ signifying absence of that medical condition. Read v2, Clinical Term Version 3 (CTV3), ICD-9 (International Classification of Diseases), and ICD-10 are alphanumeric clinical codes for describing medical conditions. Read v2 and CTV3 codes are used in primary care [25] and ICD-9 & ICD-10 in secondary care [26]. Codebases of these codes were used to classify medical conditions, ensuring primary and secondary care diagnoses were captured for each participant before the index date. All codebases are available to view in Supplementary Appendix 5. Self-reported geriatric syndromes, including dizziness, urinary frequency, and general pain or discomfort, were also captured.

### Defining the drug burden index

The DBI was computed as a binary factor, with ‘1’ signifying the presence of DBI medications and ‘0’ indicating their absence. The UK Biobank tracks participant prescriptions through linked records in the ‘gp_scripts’ entity within the UKB-RAP, using Read v2 codes, BNF codes, dm + d codes, and generic names. The R vector detailing all DBI medications can be seen in Supplementary Appendix 6.

### Modelling strategy

Random Forest (RF), XGBoost (XGB) and Logistic Regression (LR) models were validated using all 34 variables (Supplementary Appendix 3), except ethnicity, which was imbalanced. The unbalanced dataset, with fewer 30-day emergency hospitalisations than non-hospitalisations, could obscure statistical patterns and associations. We employed the Synthetic Minority Oversampling Technique (SMOTE) to increase the number of hospitalisation cases, thereby balancing the dataset and ensuring a robust analysis. Supplementary Appendix 7 shows the frequency distributions of features before and after applying SMOTE. SMOTE was applied before the train-test split, and all training and testing of the ML models were performed on the SMOTE-balanced dataset. All three models were validated using five-fold 100-repeated cross-validation. Variable importance plots represent the aggregated importance scores by factor level. Variable importance is calculated differently for different models: LR evaluates the absolute values of model coefficients, RF measures the contribution of each variable to impurity reduction across trees, and XGB assesses the impact of each variable on improving tree splits. The top 25 most important variable factor levels were validated in predicting 30-day emergency hospitalisation out of the total 69 variable factor levels. If a factor reference level (denoted with a ‘*’) appears in the plot; this signifies that this variable is important for predicting a lack of 30-day emergency hospitalisation. Information regarding the hyperparameter tuning can be seen in Supplementary Appendix 8. All models were built and evaluated in R version 4.3.0 [27]. The ML code has been deposited in a GitHub repository: github.com/RobertOlender/ML_UKBiobank_emergency_30-day_hospitalisation to enable reproducibility.

## Results

### Random Forest

**Figure 2:**
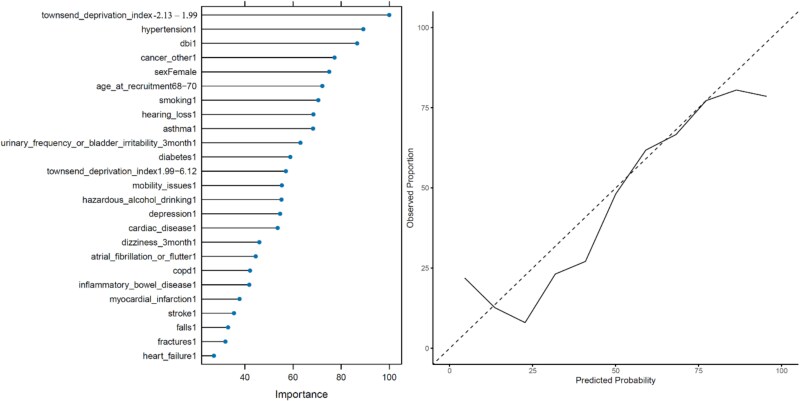
Left: Variable importance plot for the RF model. Right: Probability calibration curve for the RF model.

The RF model validated our findings from the InterRAI study, with hypertension, DBI, non-haematological/non-lung cancer, and smoking among the most important variables associated with 30-day emergency hospitalisation. Several modifiable risk factors were also validated; mobility, hazardous alcohol drinking, falls, and fractures were the 13th, 14th, 23rd, and 24th most important variables, respectively. Common geriatric syndromes urinary frequency or bladder irritability issues, and dizziness were also identified. A Townsend deprivation index between −2.13 and 1.99 was the most important risk factor. The variable importance plot and the probability calibration curve for the RF model can be seen in Figure 2. The RF model achieved an AUC-ROC of 0.785, signifying a good ability to distinguish between positive and negative 30-day emergency hospitalisation outcomes.

### XGBoost

**Figure 3:**
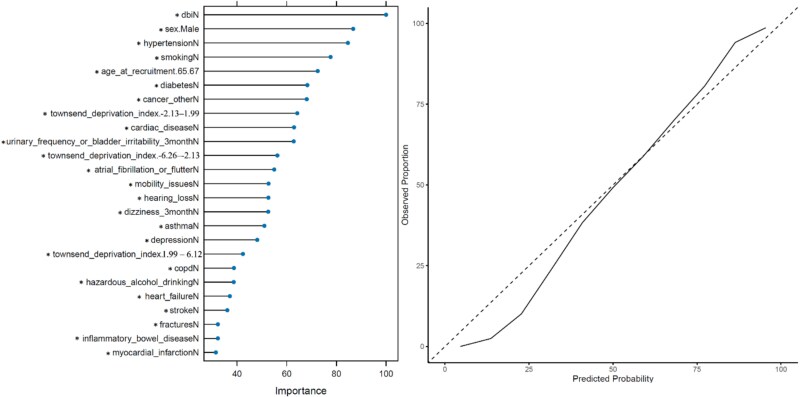
Left: Variable importance plot for the XGB model. Right: Probability calibration curve for the XGB model.

The XGB model validated our findings from the InterRAI study, with DBI, male sex, hypertension, and smoking as the four most important variables in predicting the lack of 30-day emergency hospitalisation. Like the RF model, the XGB also validated hazardous alcohol drinking, mobility, fractures, and common geriatric syndromes urinary frequency or bladder irritability issues, and dizziness as important modifiable risk factors of 30-day emergency hospitalisation. The XGB model variable importance plot and the probability calibration curve can be seen in Figure 3. The XGB model achieved a high AUC-ROC of 0.863, leading all three models, signifying a strong ability to distinguish the outcome class. While the RF and XGB models both operate through building decision trees, the XGB model iteratively corrects the error from previous trees by assigning weight to deficient variables. The XGB model favoured using the reference level (denoted with a ‘*’) to predict the lack of 30-day emergency hospitalisation.

### Logistic Regression

**Figure 4:**
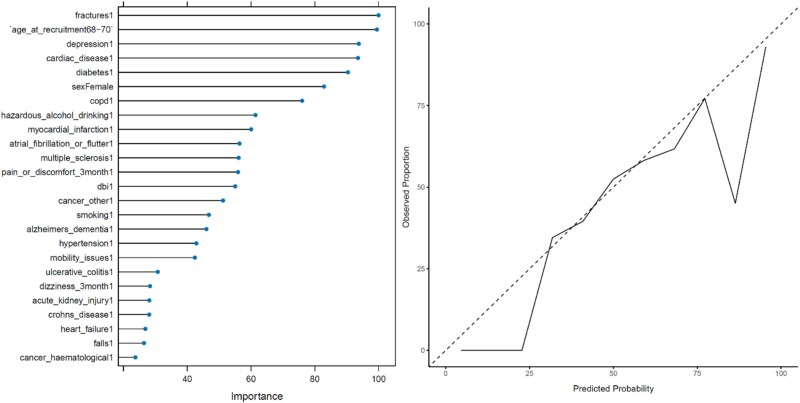
Left: Variable importance plot for the LR model. Right: Probability calibration curve for the LR model.

The LR model validated fractures, age, depression, cardiac disease and cardiac disease as the five most important variables in predicting 30-day emergency hospitalisation. Several modifiable risk factors for predicting 30-day emergency hospitalisation were validated, including hazardous alcohol drinking, DBI, smoking, mobility and falls. Considering variable importance, LR validated similar predictors of 30-day emergency hospitalisation in the InterRAI study and the UK Biobank study. The variable importance plot and the probability calibration curve for the LR model can be seen in Figure 4. The LR model achieved an AUC-ROC of 0.608. The LR model showed the lowest performance across all metrics.

### Model performance

While the aim of this study was not to develop a prediction model but rather to validate previously identified risk factors of 30-day emergency hospitalisation, a comprehensive evaluation of model performance is crucial. Table 1 shows the metrics by which the models were evaluated for both the UK Biobank and the InterRAI datasets. In the previous study using the InterRAI dataset, the RF, XGB and LR models achieved AUC-ROC scores of 0.97, 0.90 and 0.72, respectively, outperforming the current study’s models [14]. Supplementary Appendix 9 contains a list of formulas for all of the evaluation metrics. Supplementary Appendix 10 contains the confusion matrix, AUC-ROC curve and probability calibration curve for all three models using the UK Biobank. Supplementary Appendix 11 contains calibration curves after applying isotonic regression and Platt scaling [28].

**Table 1:** Model performance metrics, comparing models trained utilising the UK Biobank and InterRAI datasets.

	UK Biobank—Current study (95% CI)^a^			InterRAI (95% CI)		
	RF	XGB	LR	RF	XGB	LR
AUC-ROC	0.785 (± 0.004)	0.863 (± 0.003)	0.608 (± 0.005)	0.971 (± 0.004)	0.895 (± 0.008)	0.724 (± 0.012)
Accuracy	0.754 (± 0.004)	0.752 (± 0.004)	0.570 (± 0.004)	0.921 (± 0.006)	0.824 (± 0.009)	0.668 (± 0.011)
Balanced Accuracy	0.754 (± <0.001)	0.752 (± <0.001)	0.570 (± 0.002)	0.921 (n.a.)	0.823 (n.a.)	0.668 (n.a.)
Sensitivity	0.789 (± 0.003)	0.710 (± 0.001)	0.453 (± 0.002)	0.934 (n.a.)	0.781 (n.a.)	0.691 (n.a.)
Specificity	0.718 (± 0.003)	0.793 (± 0.001)	0.687 (± 0.002)	0.907 (n.a.)	0.864 (n.a.)	0.645 (n.a.)
PPV	0.737 (± 0.002)	0.774 (± 0.001)	0.591 (± <0.001)	0.913 (n.a.)	0.845 (n.a.)	0.672 (n.a.)
NPV	0.773 (± 0.002)	0.732 (± 0.001)	0.556 (± <0.001)	0.929 (n.a.)	0.806 (n.a.)	0.665 (n.a.)
F1 Score	0.762 (± <0.001)	0.741 (± <0.001)	0.513 (± <0.001)	0.924 (n.a.)	0.812 (n.a.)	0.681 (n.a.)

*Note*. 95% CIs are n.a. (not available) for several metrics from the InterRAI study.

^a^Model performance based on out-of-sample errors from the training set when performing repeated cross-validation. Abbreviations: PPV, positive predictive value; NPV, negative predictive value.

## Discussion

This study validated that DBI, smoking and hazardous alcohol consumption are important variables for predicting 30-day emergency hospitalisation. Smoking [29, 30] and alcohol consumption [31] are widely recognised risk factors for emergency hospitalisation, and the benefits of their cessation are well-documented [32, 33]. High DBI has been linked to poor clinical outcomes [18], and deprescribing DBI drugs has seen limited success [19]. This underscores the importance of including the DBI and other measures of potentially inappropriate polypharmacy as essential variables in future ML studies concerning older adults.

This is the first study to predict 30-day emergency hospitalisation using an RF model in older adults. The RF model achieved an AUC-ROC of 0.785 and validated several common risk factors such as DBI, sex, age, smoking and alcohol consumption. This aligns with findings from other studies that predict related clinical outcomes using RF models. For example, Veyron *et al*., developed an RF model to predict 7-day and 14-day ED visits, achieving AUC-ROC scores of 0.70 and 0.67, respectively [34]. Another study by Belmin *et al.*, predicted short-term ED visits with an RF model, achieving a sensitivity of 83% and a specificity of 86% despite a small sample size [35]. The Townsend deprivation index was the most important risk factor of 30-day emergency hospitalisation in the RF model. Since residential area deprivation is a recognised risk factor for hospitalisation [36], including it in future models may help identify high-risk individuals from deprived areas.

The XGB model achieved an AUC-ROC of 0.863 and also validated several common risk factors, including DBI, hypertension, smoking, alcohol consumption, mobility and diabetes. The performance of our XGB model is consistent with other studies, such as the one by Bories *et al*., which developed an XGB model to predict hospitalisation for bleeding events in older adults, achieving an AUC-ROC of 0.72 [37]. Another study by Polo Friz *et al*., used an XGB model to predict 30-day readmission after heart failure hospitalisation, achieving an AUC-ROC of 0.803 [38].

The LR model performed poorly, with an AUC-ROC of 0.608. This is significantly lower than the AUC-ROC of 0.724 reported in the InterRAI study. In the wider literature, LR models have shown better performance. For instance, Verdu-Rotellar *et al*., used a multivariable LR model to predict 30-day hospitalisation in older adults, achieving an AUC-ROC of 0.73. The LR model utilised clinical variables that were easily obtained in primary care settings, demonstrating good performance [39].

The current study also validated other risk factors, including mobility, fractures, and falls, which necessitate a thorough geriatric assessment, tailored therapy, and preventative measures. While mitigating these risk factors is unlikely to be impactful in the context of mitigating 30-day emergency hospitalisation risk, their validation aligns with existing literature. For instance, Fisher *et al*., identified mobility as an important variable in predicting 30-day readmission in a cohort of 111 participants aged ≥65 years hospitalised with an acute medical illness [40]. Moreover, Somersalo *et al*., showed that hip, ankle, wrist, spine and proximal humerus fractures accounted for 64% of fractures requiring hospitalisation in a cohort of 5985 participants [41]. Furthermore, in a study by Vaishya *et al*., falls were linked with hospitalisation. The group concluded that prevention by ensuring a safe living environment is more important than management [42]. Similarly to the DBI, these risk factors should be considered in future geriatric ML studies.

Validating important risk factors like DBI, smoking, and hazardous alcohol consumption for predicting 30-day emergency hospitalisation allows clinicians to mitigate the risk. This study offers a pathway for early interventions and personalised care strategies, ultimately aiming to reduce hospital admissions and improve public health in older adults. The models in this study, especially the XGB model, show promising predictive power and may be suitable for trial deployment in clinical settings upon further optimisation. The model would be deployed as a clinical decision support tool, into which a clinician would enter the patient information and obtain immediate information regarding the risk of acute hospitalisation for said patient, similar to the commonly used Fracture Risk Assessment Tool for fracture risk estimation [43]. However, despite its good performance, poor calibration in the tail-ends of the probability distributions means it is not deployable in its current form.

Several factors must be considered before deploying the XGB model in clinical settings. While the model exhibits good predictive power and calibration, future iterations should focus on optimising feature selection and more comprehensive tuning to improve the performance. All three models exhibit calibration issues at the tail-ends of the distribution, meaning that they produce less accurate predictions for low and high risk participants. Missing or poor-quality data could reduce the models’ predictive power. However, the model uses easily accessible binary data, which can be collected via simple questionnaires, without specialist training. A recent systematic review concluded that while several studies have deployed ML models in clinical settings, these studies are typically of low quality, failing to adhere to reporting standards, and failing to include participants from heterogenous populations [44].

Future work should follow standardised reporting guidelines and prioritise validating key risk factors for predicting emergency hospitalisations in diverse, generalisable older adult cohorts and calibrating models for clinical use.

This study has several limitations. The study population features a narrow age range, with over 99% of participants aged between 65 and 70 years, and concerns whether the results can be generalised to the overall ≥65 years older adult population. However, understanding health outcomes in a cohort of younger, less multimorbid older adults can provide mitigation insights applicable to older age groups. Additionally, the potential of early intervention in polypharmacy management may reduce long-term health complications and hospital admissions as patients age. Second, this cohort of volunteer participants (self-selection bias) is generally healthy and, therefore, cannot be generalised to the older adult population [45]. Third, analysis was conducted on a SMOTE-balanced dataset concerning 30-day emergency hospitalisations, which may have resulted in over-optimistic performance metrics, and may limit the generalisability to real-world scenarios. Future work will evaluate the model’s performance on unbalanced test data to assess real-world applicability. Fourth, a set index date assumes that risk factors remain stable over time, meaning that patterns observed on the index date hold for other dates. A rolling index date was not feasible to implement in this study, but should be used in future studies. Finally, ethnicity was not included in the models presented in this study because it was imbalanced (>90% British).

This study also has several strengths. Validating risk factors using new datasets gives clinicians confidence that the results are robust. Second, the variable importance plots show general agreement on key risk factors, such as DBI and hazardous alcohol drinking. Third, we captured primary and secondary care, and self-reported diagnoses. Fourth, the study differentiates between elective and emergency hospitalisation and accounts for healthy user bias by excluding patients hospitalised 1 year before the index date, increasing the clinical relevance of the findings. Fifth, the TRIPOD-AI checklist was followed, ensuring transparency across all aspects of the current study. Sixth, all ML models underwent five-fold cross-validation repeated 100 times, preventing overfitting. Finally, all R and medical code lists have been shared to enable reproducibility. The last two strengths of this study support the Findable, Accessible, Interoperable, and Reusable artificial intelligence principles to enable the reusability of scholarly data [46].

## Conclusions

An increased focus on training ML models using heterogeneous patient cohorts from multiple countries and healthcare systems may allow clinicians to utilise an ML approach to mitigate the risk of short-term emergency hospitalisations in older adults. These ML models can be applied to predict high-risk patients and identify modifiable risk factors to lower their risk of emergency hospitalisation. As demonstrated in the current study using the UK Biobank cohort and previous studies utilising InterRAI data, short-term modifiable risk factors, such as the DBI, play a crucial role in predicting emergency hospitalisations. Early recognition and mitigation of these modifiable risk factors may lead to lowering the patient’s risk and improving the standard of clinical care and public health. It may also provide a foundation for future researchers to include them in their predictive models.

## Supplementary Material

aa_24_2377_File002_afaf156

## Data Availability

More details concerning the UK Biobank and how to access the datasets can be found at www.ukbiobank.ac.uk/. The analytical code has been deposited in a GitHub repository: github.com/RobertOlender/ML_UKBiobank_emergency_30-day_hospitalisation to enable reproducibility.
